# TRP drop, TRP drop: a steady patter of anti-schistosomal target illumination

**DOI:** 10.3389/fpara.2024.1349623

**Published:** 2024-02-13

**Authors:** Daniel J. Sprague, Claudia M. Rohr, Jonathan S. Marchant

**Affiliations:** Department of Cell Biology, Neurobiology & Anatomy, Medical College of Wisconsin, Milwaukee, WI, United States

**Keywords:** parasite, schistosomiasis, TRP channels, praziquantel, meclonazepam

## Abstract

Infections caused by parasitic flatworms impart a significant disease burden. This is well exemplified by the neglected tropical disease schistosomiasis, which afflicts millions of people worldwide. The anti-schistosomal activity of various chemotypes has been known for decades, but the parasite targets of many of these remain undefined. Until recently, this included the current clinical therapy, praziquantel (PZQ). However, the tempo of target discovery has recently gathered pace, with discoveries of schistosome targets for praziquantel (PZQ) and the anthelmintic benzodiazepine, meclonazepam (MCLZ). This steady patter of target illumination has also revealed a pattern in that both PZQ and MCLZ target members of the same ion channel subgroup—transient receptor potential ion channels of the melastatin family (TRPM channels). PZQ activates one member of this family (TRPM_PZQ_) and MCLZ activates a different channel (TRPM_MCLZ_). Here, similarities and differences between these two new targets are discussed. These data highlight the need for further study of TRPM channels in parasitic flatworms given their vulnerability to chemotherapeutic attack.

## 1 Introduction

Compounds that are active against parasitic flatworms (disease-causing flukes and tapeworms) have been known for a long time (Keeling, 1968; Lammler, 1968; Katz, 1977). However, our understanding of the mechanism of action of many of these anthelmintics has long been proven incomplete as their targets within the parasite lack definition.

**Figure 1:**
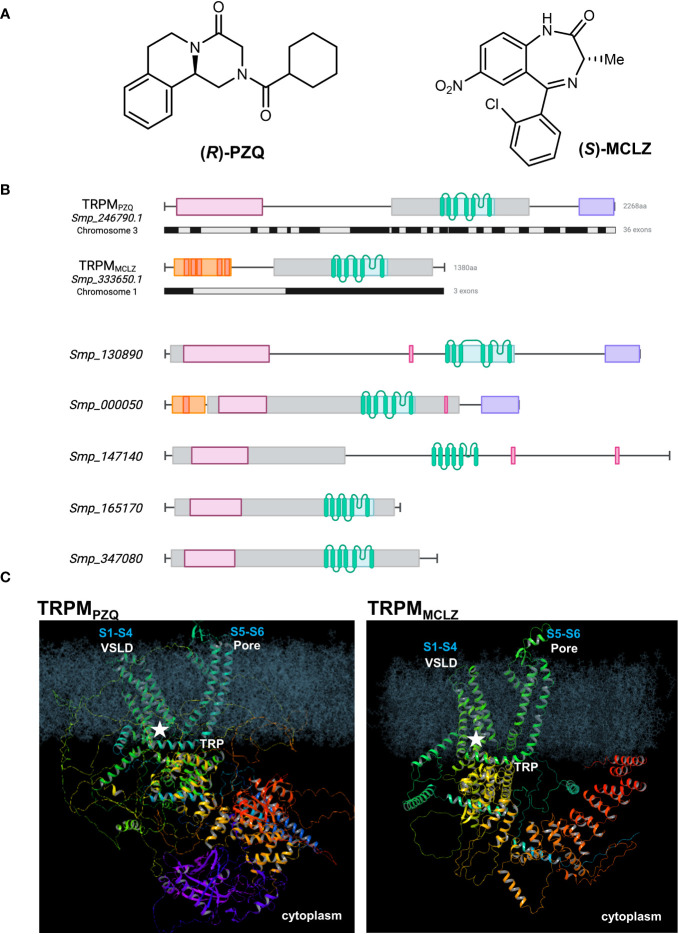
Praziquantel and meclonazepam target distinct schistosome TRPM channels. **(A)** Chemical structures of (*R*)-PZQ (left) and (*S*)-MCLZ (right), activators of *Sm*.TRPM_PZQ_ and *Sm*.TRPM_MCLZ_, respectively. **(B)** *Top*, schematic overview of *Sm*.TRPM_PZQ_ (transcript, *Smp_246790.1*) and *Sm*.TRPM_MCLZ_ (transcript, *Smp_333650.1*). Exon organization of each ion channel is shown below the protein domain schematic (black, grey). *Bottom*, schematic organization of other schistosome TRPM paralogs. Annotated domains are shown as follows: TRPM/SLOG domain (pink), PTHR13800 (a TRPM family curation, gray), transmembrane helices (green), NUDIX hydrolase (purple), and ankyrin repeat domain (red and orange) with discrete repeats. Genomic identifiers are from v18 of WormBase Parasite (Howe et al., 2017). The prior transcript identifier for *Sm*.TRPM_PZQ_ was Smp_246790.5 in v17. **(C)** Model of *Sm*.TRPM_PZQ_ (left) and *Sm*.TRPM_MCLZ_ (right) embedded in a lipid bilayer depicting the overall predicted structure and the location of the binding site (star) for each drug within the VSLD cavity (S1–S4) of the ion channel. The pore domain (S5–S6) and the TRP helix are also shown. Models were generated from AlphaFold (Varadi et al., 2022) and depict a single monomer of each ion channel.

The current clinical therapy for schistosomiasis, praziquantel (PZQ, **Figure 1A**), provides a very good example. Discovered by Bayer AG and Merck-KGaA in the early 1970s, it has been available for clinical use since 1978 (King and Mahmoud, 1989). However, PZQ has lacked definition of a target in schistosomes for over 40 years (Park et al., 2019; Park and Marchant, 2020; Brunetti et al., 2021). This lack of knowledge reflects the challenges inherent to target identification, especially using parasite models, where key methods are often not available or difficult to execute. The lack of target validation is a frustrating barrier for rational drug design. Deciphering a target mechanism is a spur to drug development, facilitating target-based screening campaigns, leading to optimization and subsequent improvement of the drug candidate through the preclinical pipeline. Further, knowledge of a target can provide a better understanding of worm biology, illuminating additional targets upstream and downstream in the relevant signaling pathway. Finally, knowledge of the drug target facilitates surveillance for drug resistance. While resistance mechanisms are multifactorial, one obvious site for resistance is polymorphism within the drug target itself where, for example, variation within the ligand binding site can confer drug insensitivity (Glickman and Sawyers, 2012). Target-driven resistance to antifolate drugs used for malarial chemotherapy provides a well-known example (Cowell and Winzeler, 2019).

The benzodiazepine, meclonazepam (MCLZ, **Figure 1A**), patented in 1977 by Hoffman La Roche is another example of an old compound with anti-schistosomal activity (Stohler, 1979). MCLZ, like PZQ, causes schistosome paralysis and surface damage (Pax et al., 1978; Bricker et al., 1983). Also, like PZQ, MCLZ has long lacked definition of a molecular target. While MCLZ is an enticing anti-schistosomal chemotype given its efficacy against juvenile worms (Pica-Mattoccia et al., 2008), it cannot progress as a clinical drug owing to host side effects including sedation and psychomotor depression (Baard et al., 1979). These effects are caused by benzodiazepine action on host GABA_A_ channels in the central nervous system, resulting in a narrow therapeutic window (Baard et al., 1979). More broadly, MCLZ is a ‘designer benzodiazepine’ (Brunetti et al., 2021) and a drug of abuse. Identification of the parasite target of MCLZ would provide an opportunity to engineer away the determinants responsible for these detrimental host activities, a hope that has kindled recurring interest in this ligand over several decades.

Recent work has now unmasked parasite targets for both PZQ (Park et al., 2019) and MCLZ (Park et al., 2024), providing defined candidates for further investigation. Both targets are ion channels within the same subfamily, the melastatin family of transient receptor potential ion channels, known as ‘TRPM’ channels. PZQ activates a TRPM channel [named TRPM_PZQ_ (Park et al., 2019; Marchant, 2024)], and MCLZ activates a different TRPM family member [named TRPM_MCLZ_ (Park et al., 2024)]. These discoveries now catalyze the opportunities enabled by target identification to be realized.

In this mini-review, we highlight similarities (Section 2) between these TRPM channel targets, as well as some differences (Section 3) that could be important for deciphering the roles of these ion channels in schistosome biology. The discussion (Section 4) identifies opportunities for future work.

## 2 Similarities

### 2.1 Similar ion channel family

The targets of PZQ and MCLZ are both ion channels. Both are TRP channels. Both are siblings within the same TRPM subfamily (**Figure 1B**). This clade of ion channels therefore emerges as a class of targets with an enticing chemotherapeutic vulnerability.

In humans, TRPM channels serve as polymodal sensors that respond to a broad diversity of stimuli and environmental cues (Huang et al., 2020). They can be activated by changes in temperature, osmolarity, and oxidative stress, as well as by phytochemicals, endogenous mediators, and various classes of synthetic ligands. The eight human TRPM channels (hTRPM1–8) have diverse functions, playing roles in Ca^2+^ and Mg^2+^ homeostasis, thermosensation, secretion, cell migration, inflammation, immunomodulation, and cell adhesion (Huang et al., 2020). These channels are being scrutinized as therapeutic targets in multiple disease states and are the focus of various drug discovery efforts (Koivisto et al., 2022).

Diversification of TRPM channels has occurred independently within the lophotrochozoan lineage, distinct from the vertebrate TRPM1–8 expansion (Burroughs et al., 2015; Zajac et al., 2021). As such, the pharmacological sensitivities of parasitic flatworm TRPM channels will likely prove unique, presenting opportunities for selective targeting. This has fortuitously proved the case with PZQ which exhibits relatively few side effects on the human host. Targeting TRPM channels in parasitic flatworms therefore seems a viable strategy bolstered by the recent discovery of TRPM_PZQ_ and TRPM_MCLZ_.

### 2.2 Same binding pockets

Both PZQ and MCLZ are TRPM agonists and both engage their TRPM targets through the same ligand binding site. This binding pocket is formed from the first four transmembrane helices (S1–S4) and the TRP helix of the ion channel, within the voltage–sensor-like domain (VSLD) cavity (**Figure 1C**). The agonists of both parasitic flatworm TRPM channels retain broadly similar physiochemical properties (size, hydrophobicity, and chemical space) and exploration of their structure–activity relationships has revealed stringent requirements for agonism (Menezes et al., 2012; McCusker et al., 2019; Park et al., 2021; Sprague et al., 2023). These stringent requirements are imposed by architectural determinants of the VSLD binding pocket. This VSLD binding site resembles the ligand binding pocket found within the VSLD of the human TRPM8 (*Hs*.TRPM8) channel, which, in vertebrates, can accommodate a broad variety of chemotypes (Gonzalez-Muniz et al., 2019). The structure of the *Hs*.TRPM8 pocket in complex with various agonists and antagonists has been elaborated in multiple structural studies over the last decade (Huang et al., 2020), and conservation with the architecture of the parasite TRPM binding pockets is evident (Park et al., 2021). This has been demonstrated through modeling and functional profiling following mutagenesis of conserved residues (Park et al., 2021).

### 2.3 Similar functions

**Figure 2:**
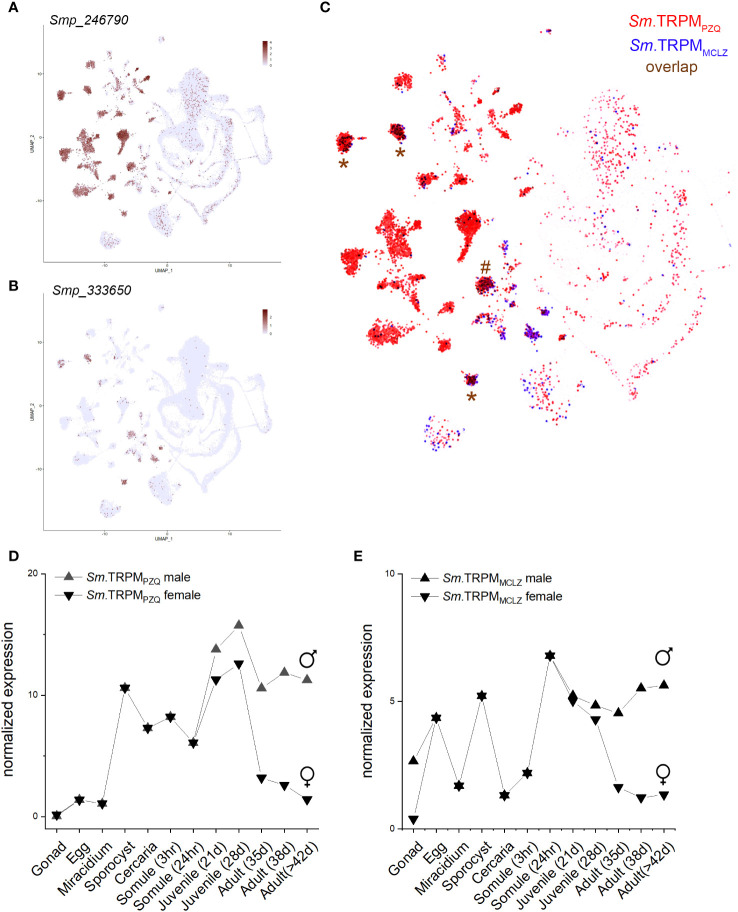
Expression profile of *S. mansoni* TRPM_PZQ_ and TRPM_MCLZ_. Data were from the schistosome single-cell transcriptome atlas for **(A)** *Sm*.TRPM_PZQ_ (*Smp_246790*) and **(B)** *Sm*.TRPM_MCLZ_ (*Smp_333650*) with overlap highlighted in **(C)**. Data were downloaded from the Collins lab SchistoCyte Atlas (Wendt et al., 2020; Wendt et al., 2021). Cell clusters where the channels show considerable overlap are highlighted for three neuronal (*) and a muscle cluster (#). **(D, E**) Lifecycle stage expression data for mixed-sex infections for **(D)** *Sm*.TRPM_PZQ_ and **(E)** *Sm*.TRPM_MCLZ_. Data were downloaded (v7) and replotted from the Berriman Lab gene expression portal (Lu et al., 2018).

Both drugs cause cellular depolarization, and both TRPM_PZQ_ and TRPM_MCLZ_ show little evidence for desensitization in response to PZQ or MCLZ under optimized recording conditions. Single cell RNA sequencing data revealed both channels are expressed in excitable cells in adult worms (**Figures 2A, B**), such that channel activation would be expected to cause a protracted exocytosis (nerves) and contraction (muscle). PZQ has been shown to activate a native TRPM_PZQ_-like endogenous ion channel blocking endogenous oscillations observed in motor neurons (Chulkov et al., 2023). Both drugs cause spastic muscle contraction, and both damage the tegument. That these grossly similar effects (depolarization, muscle contraction, and tegument damage) are similar for both drugs is perhaps unsurprising given that their targets display similar properties and tissue expression patterns (**Figure 2C**). Both channels are expressed throughout the parasitic lifecycle (Lu et al., 2018), although given the signal amplification inherent to ion channel action, mRNA levels of these TRPM channels are low (**Figures 2D, E**). In hindsight, it is both obvious and reassuring that two drugs with grossly similar phenotypic outcomes (muscle contraction, depolarization, and surface damage) share a similar mechanism of action (TRPM agonists). This prompts the question as to whether other drugs that cause similar phenotypes also act as TRPM ligands.

## 3 Differences

### 3.1 Architecture

TRPM_PZQ_ and TRPM_MCLZ_ are distinct ion channels, differing in their size and architecture. First, in terms of genomic organization (Howe et al., 2017), the gene encoding *Sm*.TRPM_PZQ_ (*Smp_246790*, chromosome 3) spans ~150 kb and comprises 36 exons. TRPM_MCLZ_ is encoded by three exons within a gene spanning ~50 kb (*Smp_333650*, chromosome 1; **Figure 1B**). This difference clearly affords the possibility for a greater diversity of splice variants of *Sm*.TRPM_PZQ_. The transcripts used for functional profiling of each TRPM channel encode proteins of 2,268 amino acids (*Sm*.TRPM_PZQ_, ~250 kDa) and 1,380 amino acids (*Sm*.TRPM_MCLZ_, ~150 kDa). Assuming each TRPM monomer assembles as a tetramer, the two TRPM channel complexes differ in size (~1 MDa vs. ~600 kDa).

The modular organization of these two TRPM channels is also distinct. The cytoplasmic, COOH-terminal region of *Sm*.TRPM_PZQ_ contains a nudix domain homologous to the mitochondrial ADP ribose pyrophosphatase NUDT9 [NUDT9H (Perraud et al., 2001)], that in vertebrate TRPM2 channels binds adenosine diphosphate ribose (ADPR). Nothing is currently known about the binding properties or specificity of this domain in TRPM_PZQ_. The enzymatic capacity of this domain in TRPM_PZQ_ is also unknown. Investigating these unknowns could be of interest given the unique purine and pyrimidine metabolic pathways in schistosomes (El Kouni, 2017; Skelly et al., 2022) and the potential sensitivity of schistosomes to oxidative stress (Huang et al., 2012). *Sm*.TRPM_MCLZ_ has a shorter cytoplasmic COOH terminal region, lacking any enzyme domain (Himmel et al., 2020).

The N-terminal cytoplasmic domain of both TRPM channels also differs. TRPM channels have traditionally been defined by the presence of homologous NH_2_-terminal regions [‘TRPM homology region’, MHR, structured as MHR1/2, MHR3, and MH4 domains (Huang et al., 2020)]. The TRPM NH_2_ terminal regions display sequence homology and topological homology with the SMF/DprA-LOG (SLOG) superfamily found in bacteria and plants (Himmel et al., 2020). This MHR/SLOG organization is characteristic of the TRPM family compared with other TRP subfamilies (Huang et al., 2020). Whereas TRPM_PZQ_ displays this distinctive TRPM architecture, TRP_MCLZ_ lacks the NH_2_-terminal MHR1/2 or SLOG domain (**Figures 1B, C**). Rather, the NH_2_ terminus of TRPM_MCLZ_ contains modules reminiscent of ankyrin-repeats, which are found downstream of the SLOG domain in TRPM channels but are more commonly associated with the TRPA, TRPC, TRPN, and TRPV channel subfamilies.

Finally, the channel pore domains may differ, as, based on sequence comparison of the two channels, conservation appears low. Future electrophysiological studies of TRPM_MCLZ_ will be needed to characterize the cation selectivity of TRPM_MCLZ_ compared with TRPM_PZQ_.

### 3.2 Ligand selectivity

As discussed earlier, PZQ and MCLZ engage the same VSLD ligand binding pocket in their respective TRPM targets. However, the architecture of the two binding pockets is distinct as both channels show distinct ligand binding specificities: PZQ does not activate TRPM_MCLZ_ and reciprocally, MCLZ does not activate TRPM_PZQ_ (Park et al., 2024). One contributing factor is the identity of an acidic residue within the channel TRP domain, that acts as a gatekeeper residue for the VSLD pocket (Rohr et al., 2023). In TRPM_PZQ_, this residue is an aspartic acid residue that does not impair PZQ occupancy of the VSLD binding site. However, in the other schistosome TRPM channels, including TRPM_MCLZ_, this residue is a glutamic acid that appears non-permissive of PZQ occupancy of the VSLD pocket impairing PZQ association (Rohr et al., 2023). Whether other TRPM channels interact with MCLZ will require further investigation, and clearly, differences between the TRPM ortholog binding pockets will dictate the breadth of chemotypes accommodated. Whether the pockets of TRPM_PZQ_ and TRPM_MCLZ_ are completely exclusive of ligands capable of engaging their sibling’s binding pocket will require investigation. We note that high concentrations of MCLZ inhibited PZQ activation of TRPM_PZQ_, suggesting some overlap (Park et al., 2024). This possibility is intriguing, as identification of an agonist able to engage both channel binding pockets would minimize the likelihood of drug resistance, as the chances of dual resistance mutations emerging simultaneously at both targets to block drug action would be low.

Whereas PZQ shows a broad spectrum activity against parasitic flatworms (with the exception of *Fasciola* spp.), MCLZ possesses a more restricted scope of action. For example, it is not effective against all species of schistosomes, being poorly effective against *S. japonicum* (Pax et al., 1978; Stohler, 1979; Pica-Mattoccia et al., 2008). The likely explanation is again a target polymorphism—the lower sensitivity of *S. japonicum* TRPM_MCLZ_ to MCLZ is explained by a naturally variant binding pocket residue that sterically impedes MCLZ occupancy in *Sj*.TRPM_MCLZ_ (Park et al., 2024). This residue (a tyrosine at the base of the S4 helix, Y944 in *Sj*.TRPM_MCLZ_) appears conserved throughout the clade of ‘Asian’ [the *S. japonicum* clade (Lawton et al., 2011)] versus ‘African’ schistosomes (the *S. mansoni* and *S. haematobium* clades), suggesting that MCLZ would show poor efficacy against other schistosome species (Park et al., 2024).

### 3.3 Activity against juvenile worms

Drug efficacy against juvenile worms (typically assessed ≤5 weeks after infection) is a very appealing characteristic for an anti-schistosomal therapy. Activity against immature worms maximizes the likelihood of a curative outcome from a single dose treatment. This property is a feature of MCLZ, but not PZQ (Pica-Mattoccia and Cioli, 2004), even though PZQ [EC_50_ of ~300 nM for (*R*)-PZQ (Rohr et al., 2023)] shows a higher sensitivity at TRPM_PZQ_ than MCLZ at TRPM_MCLZ_ [EC_50_ of ~1 µM for (*S*)-MCLZ (Park et al., 2024)]. Could a critical difference between TRPM_MCLZ_ and TRPM_PZQ_ be the ability of TRPM_MCLZ_ activators, but not TRPM_PZQ_ activators, to confer activity against juvenile worms? The different channels could engage different downstream signaling pathways, be expressed at different levels, or be present within cell subpopulations with different essentiality to the viability of young versus adult schistosomes. Whatever the explanation, these possibilities should be investigated. If TRPM_MCLZ_ engagement proves to be intrinsically schistosomicidal, there would be a compelling case to discover new chemotypes active at TRPM_MCLZ_, or improve versions of currently realized activators that are not feasible therapeutics (Park et al., 2024).

However, jumping to such a conclusion would be premature, as this suggestion is based on the properties of only a single activator of each channel (PZQ versus MCLZ). It is also possible that activity against juvenile worms may relate to ligand pharmacokinetic and pharmacodynamic (PK/PD) considerations that define the time course of worm exposure to the different drugs (Abla et al., 2017). PZQ is metabolized much more rapidly than MCLZ [half-life of PZQ ≤5 h compared with ~40–80 h for MCLZ, respectively (Vikingsson et al., 2017; Kovac et al., 2018)]. So, while nurturing the idea that a key difference between these new targets is that TRPM_MCLZ_ activators uniquely confer lethality toward juvenile worms, further investigation of other chemotypes and their PK/PD properties is warranted.

## 4 Discussion

The recent identification of different parasitic flatworm TRPM channels activated by PZQ (Park et al., 2019) and MCLZ (Park et al., 2024) underscores the relevance of the TRPM subfamily of ion channels as druggable targets. This provides several opportunities moving forward.

First, both TRPM_PZQ_ and TRPM_MCLZ_ are conserved within the genomes of other parasitic flatworms (Park et al., 2019; Park et al., 2024). This provides an opportunity to design TRPM activators with broad-spectrum anthelmintic activity, as well as ligands tailored for specific infections. With both these TRPM targets in hand, this presents a wealth of target-based screening opportunities. For PZQ, progress has recently been made through the identification of other TRPM_PZQ_ ligands (Chulkov et al., 2021), and the discovery of a broad-spectrum activator of fluke TRPM_PZQ_ (Sprague et al., 2023), which, unlike PZQ, is active against *Fasciola hepatica* TRPM_PZQ_ (*Fh*.TRPM_PZQ_). PZQ has long been known to lack efficacy for treating fascioliasis, and TRPM_PZQ_ from *Fasciola* species is not activated by PZQ (Park et al., 2021; Rohr et al., 2023). Target-based design advanced a benzamidoquinazolinone ligand, BZQ, which displayed efficacy against both the *Fh*.TRPM_PZQ_ ion channel and *Fasciola hepatica* worms *ex vivo* (Sprague et al., 2023). This provides a clear example of how novel anthelmintic chemotypes can be realized by target-based screening, underscoring the importance of deorphanizing anthelmintics to identify their targets. For MCLZ, its efficacy against other trematodes and cestodes is unclear, and the properties of TRPM_MCLZ_ orthologs in other parasites have yet to be examined. This may provide an opportunity to identify new active molecules following the precedent established for BZQ at TRPM_PZQ_.

Second, there is an opportunity to study the other TRPM paralogs. Here, it is important to acknowledge that TRPM channels exhibit a deep evolutionary phylogeny. Their ancient evolutionary pedigree and the retention of a transmembrane VSLD pocket in parasitic flatworms may prove an intrinsic vulnerability to chemotherapeutic attack. The schistosome TRPM family has five additional members that have been annotated but are yet to be deorphanized (**Figure 1B**). Two of these TRPM channels contain a COOH-terminal nudix homology domain like TRPM_PZQ_ (the ‘nudix’ subclade), and the other paralogs (apart from TRPM_MCLZ_) contain the NH_2_-termnial ‘TRPM/SLOG’ plus ‘ion channel’ architecture defined in the ancestral TRPM channel (Burroughs et al., 2015). What ligands do these TRPM channels engage? How is each endogenously activated? Human TRPM channels display considerable diversity in their properties compared to the other TRP subfamilies—is the functional repertoire of parasitic flatworm TRPMs just as broad? Understanding the properties of TRPM_PZQ_ and TRPM_MCLZ_, as well as the remaining parasitic flatworm TRPM paralogs, now becomes a priority. Future studies to ablate TRPM_PZQ_ and TRPM_MCLZ_ activities through genetic and pharmacological loss-of-function manipulations would also be informative.

Finally, these data also provide the impetus for scrutiny of other schistosome TRP channel subfamilies [TRPC (four representatives), TRPP (two representatives), and TRPA and TRPML (one representative each) (Bais and Greenberg, 2020)]. From this portfolio, it has already been shown that the schistosome TRPA channel regulates motor activity [*Sm*.TRPA (Bais et al., 2015; Bais et al., 2018)] while the schistosome TRPML channel regulates neuromuscular activity and is required for tegumental integrity [*Sm*.TRPML (Bais et al., 2022)]. However, there is clearly an opportunity to profile the remaining TRPC, TRPP, and TRPM targets, and accumulate a more detailed understanding of their pharmacological specificities through target-based screening.

Similarly, TRP channels will likely also prove to be productive targets in parasitic nematodes (Choudhary et al., 2022) and other eukaryotic pathogens (Wolstenholme et al., 2011). We note that the anti-filarial drug diethylcarbamazine (DEC) has been revealed as a TRPC-like channel activator in *Brugia malayi* [*Bm*.TRP-2, (Verma et al., 2020; Williams et al., 2023)]. Activation of *Bm*.TRP-2 in muscles causes a rapid paralysis of microfilariae as well as adult worms. RNA interference targeting a different *Brugia* TRP channel, the TRPV-like channel *osm-9*, revealed a chemosensory role for this TRP channel [*Bp*.OSM-9, (Wheeler et al., 2020)], supporting the directional migration of infective L3 larvae toward serum. Inhibition of a TRP channel in *Toxoplasma gondii* impaired parasite invasion and egress [*Tg*.TRPPL-2 (Marquez-Nogueras et al., 2021)]. An intracellular TRP channel regulates subcellular iron transport in *Trypanosoma brucei* [*Tb*.MLP (Taylor et al., 2013)]. Knowledge of the functional roles of specific TRP channels in different parasites therefore continues to accrue, complementing additional insight derived from studies of TRP channel function in free-living flatworms and free-living nematodes. Understanding the contributions of each TRP channel to parasite sensory physiology, growth, and homeostasis will help guide the prioritization of targets within the TRP channel superfamily for future drug development. Hopefully, there is much promise yet to be realized.

## Author contributions

DS: Writing – review & editing. CR: Writing – review & editing. JM: Writing – original draft.
